# Prevalence of Gram-negative bacilli resistance in adult critically ill patients at admission screening

**DOI:** 10.1186/cc9645

**Published:** 2011-03-11

**Authors:** D Kotwinski, R Batra, T Olga, J Edgeworth, D Wyncoll, M Shankar-Hari

**Affiliations:** 1 King's College London & Guy's and St Thomas' NHS Foundation Trust, London, UK

## Introduction

Nosocomial infections in critically ill patients are increasing and they are often due to multidrug-resistant Gram-negative bacilli (GNB). Emerging resistance in common nosocomial pathogens is usually related to local antibiotic use. Gentamicin is the first-line empiric antibiotic for hospital-acquired infections in St Thomas' Hospital ICUs. No decontamination therapy for GNBs is employed, but rectal and nose swabs are routinely taken from patients on admission to screen for resistance in GNB. This informs the choice of antimicrobial therapy in the event of nosocomial infection during the patient's stay. We describe antibiotic resistance rates in GNB isolates at admission in critically ill adult patients over 8 years.

## Methods

An 8-year retrospective observational cohort study using prospectively collected data in a 30-bed referral ICU. *Patients*: The cohort inclusion criterion was defined as patients admitted to the ICU sat St Thomas' Hospital and remaining in the ICU for more than 24 hours. In addition, the cohort inclusion was restricted to the first admission from each patient over the 8-year period where the length of stay was greater than 24 hours and the admission screen had been conducted within the first 2 days of admission. *GNB screening*: In patients admitted to the ICU, rectal and nose swabs were sent at admission for microbiological evaluation antibiotic resistance in GNB.

## Results

Of the 8,095 ICU admissions, 4,753 patients satisfied the inclusion criteria. The case-mix characteristics and out come did notshow any statistically significant difference during the study period. Overall, the number of patients presenting with gentamicin-resistant GNBs on admission has remained stable, although time trends depend on the bacterial genus considered (9.3% in 2002 to 8.4% in 2009). Hospital-associated (ICU admission >48 hours following hospital admission) gentamicin resistance in GNB has fallen (14.8% in 2002 to 8.3% in 2009). Patients with a positive admission screen are more likely to have the same resistant genus isolated from a nosocomial infection during the same admission spell, as compared with those negative on admission.

## Conclusions

Screening for GNB resistance guides empiric antibiotic therapy.

